# Personalized prediction of pathological complete response in breast cancer neoadjuvant therapy: a nomogram combining quantitative MRI biomarkers and molecular subtypes

**DOI:** 10.3389/fonc.2025.1669700

**Published:** 2025-09-25

**Authors:** Zhendong Shi, Xiaoxing Bian, Hanyan Zhu, Chunyan Li, Jie Meng, Xiaomin Qian, Peng Zhou, Jin Zhang

**Affiliations:** ^1^ The Third Department of Breast Cancer, Tianjin Medical University Cancer Institute and Hospital, National Clinical Research Center for Cancer, Tianjin, China; ^2^ Key Laboratory of Cancer Prevention and Therapy, Tianjin Medical University Cancer Institute and Hospital, Tianjin, China; ^3^ Tianjin’s Clinical Research Center for Cancer, Tianjin Medical University Cancer Institute and Hospital, Tianjin, China; ^4^ Key Laboratory of Breast Cancer Prevention and Therapy, Tianjin Medical University, Ministry of Education, Tianjin, China; ^5^ Department of Medical Laboratory, School of Medical Technology, Tianjin Medical University, Tianjin, China

**Keywords:** breast cancer, neoadjuvant therapy, pathological complete response, magnetic resonance imaging, nomogram

## Abstract

**Purpose:**

In this study, we aimed to determine the diagnostic performance of MRI in assessing neoadjuvant therapy (NAT) response, investigate determinants of its accuracy, and develop a nomogram for predicting pathological complete response (pCR) following NAT.

**Methods:**

A retrospective analysis was conducted on 554 female patients who received NAT between January 2019 and December 2022 and underwent MRI scans pre- and post-treatment. Clinicopathological and MRI characteristics were collected. Univariable logistic regression identified predictors of diagnostic accuracy. Patients were then randomly allocated to training (n=388, 70%) and validation (n=166, 30%) cohorts. Using multivariable logistic regression in the training cohort, we identified independent predictors of pCR and constructed a predictive nomogram. Model performance was assessed in both cohorts using receiver operating characteristic (ROC) curves, area under the curve (AUC), and goodness-of-fit tests.

**Results:**

The overall accuracy of breast MRI in evaluating NAT response was 77.44%. Multivariable analysis identified three factors independently associated with reduced MRI accuracy: ER-negative status, absence of ductal carcinoma *in situ* (DCIS), and coexistence of mass lesions with non-mass enhancement (NME). Independent predictors of pCR included: ER-negative, HER2-positive, without the presence of DCIS, the coexistence of mass lesions and NME on pre-NAT MRI, radiologic complete remission (rCR), smaller tumor size, and increasing/plateau TIC on post-NAT MRI. The predictive nomogram demonstrated robust discrimination, with AUC values of 0.894 (95% CI: 0.857–0.932) in the training cohort and 0.888 (95% CI: 0.841–0.935) in the validation cohort.

**Conclusion:**

Breast MRI accuracy was reduced in ER-negative tumors, those lacking DCIS, and lesions exhibiting coexistent mass and NME. A clinicopathological-MRI integrated nomogram demonstrated robust predictive performance for pCR after NAT completion, potentially aiding in surgical strategy planning.

## Introduction

According to the latest global cancer statistics in 2022, breast cancer has become the most common malignant tumor and the leading cause of cancer-related death among women worldwide (1). Neoadjuvant therapy (NAT), which refers to systemic drug treatment before surgery, is increasingly being applied not only to patients with locally advanced disease who are initially inoperable but also to those with early-stage disease. NAT confers several advantages, including enhancing tumor resectability and augmenting the likelihood of breast-conserving surgery. Additionally, NAT response patterns enable personalized adaptation of post-neoadjuvant treatment algorithms (2). Previous research indicated that patients achieved pathological complete response (pCR) after NAT tend to have significantly better long-term outcomes (3). Some patients may even be candidates for de-escalation of treatment (4). However, a subset of patients demonstrates suboptimal response or disease progression during or following NAT, mandating timely treatment strategy adjustment to avoid ineffective therapeutic exposure. Consequently, early and precise assessment of NAT efficacy is critical for implementing individualized precision oncology paradigms.

Although pCR represents the reference standard for post-treatment tumor response assessment, its determination is inherently delayed—requiring completion of neoadjuvant therapy and subsequent surgical resection. Currently, various methods, including physical examination, breast ultrasound (US), mammography (MMG), breast magnetic resonance imaging (MRI), and positron emission tomography-computed tomography (PET-CT), are employed to assess NAT efficacy. However, none of these methods achieve the desired level of accuracy. Among them, breast MRI, particularly dynamic contrast-enhanced MRI (DCE-MRI) and diffusion-weighted MRI (DWI-MRI), demonstrates considerable potential for assessment. DCE-MRI can reflect changes in tumor blood perfusion and vascular permeability, while DWI-MRI provides information on tumor cell structure and membrane integrity, thereby endowing MRI diagnosis with high sensitivity and specificity. Despite reported overall accuracy rates of 76–90% for breast MRI in evaluating neoadjuvant therapy response, persistent diagnostic inaccuracies remain a clinical challenge (5, 6).

Previous studies have explored factors influencing the accuracy of breast MRI in assessing NAT efficacy (7, 8), but these studies were limited by small sample sizes and incomplete inclusion of factors, resulting in restricted conclusions. The present study aims to conduct a comprehensive multifactorial analysis of determinants influencing NAT response and develop a validated prediction model integrating breast MRI features with clinicopathological indicators. The visualized nomogram may provide clinicians with a precision medicine tool for early efficacy assessment and evidence-based therapeutic decision optimization.

## Materials and methods

### Study population

**Figure 1:**
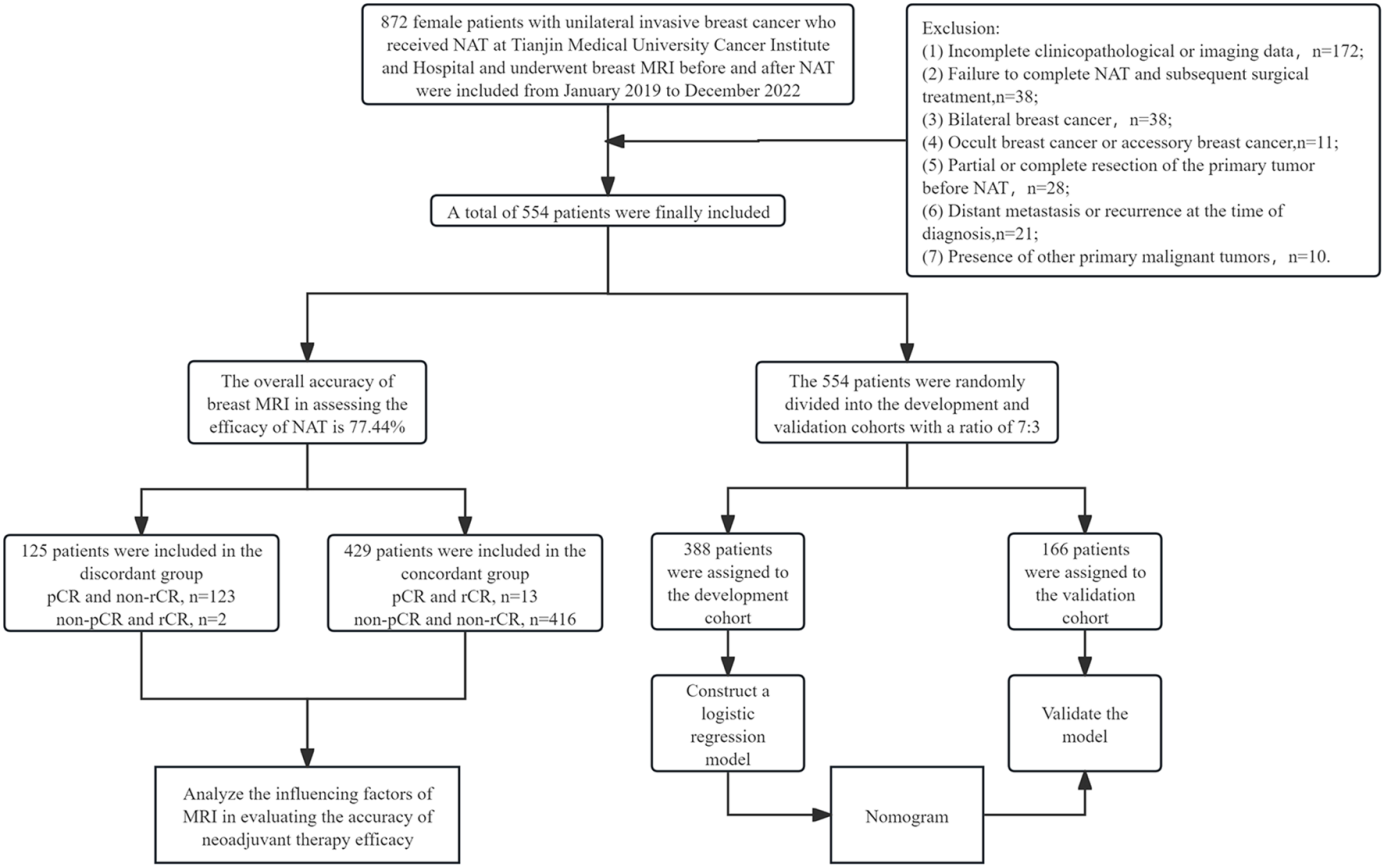
Study flowchart of the patient enrollment.

A total of 554 female patients with breast cancer who received treatment at Tianjin Medical University Cancer Institute and Hospital from January 2019 to December 2022 were ultimately included in this study. All patients had histologically confirmed invasive breast carcinoma via core needle biopsy and received guideline-concordant neoadjuvant therapy followed by definitive surgery. In addition, all patients underwent breast MRI examinations before and after NAT. The exclusion criteria were as follows: (1) incomplete clinicopathological or imaging data; (2) failure to complete NAT and subsequent surgical treatment; (3) bilateral breast cancer; (4) occult breast cancer or accessory breast cancer; (5) partial or complete resection of the primary tumor before NAT; (6) distant metastasis or recurrence at the time of diagnosis; (7) presence of other primary malignant tumors (**Figure 1**).

### Clinical characteristics

Clinical data, including age at diagnosis, menopausal status, presence of lymph node metastasis, clinical stage of the tumor, NAT regimen and cycles, and type of breast surgery (mastectomy or breast-conserving surgery), were collected. The clinical stage of the tumor was strictly determined according to the 8th edition of the American Joint Committee on Cancer (AJCC) TNM staging manual for breast cancer. All patients received standard NAT regimen before surgery. Specifically, for patients with human epidermal growth factor receptor 2 (HER2)-positive breast cancer, treatment regimens included trastuzumab monotherapy or dual-target therapy with trastuzumab and pertuzumab. For patients with hormone receptor (HR)-positive or triple-negative breast cancer (TNBC), the majority received neoadjuvant therapy regimens based on anthracycline-based chemotherapy combined with or followed by taxane-based chemotherapy.

### Histopathologic analysis

Pathological parameters were evaluated using pretreatment core needle biopsy specimens and definitive surgical resection specimens, including: histological type, presence of DCIS, histological grade, molecular subtype, expression of estrogen receptor (ER), progesterone receptor (PR), HER2, Ki-67, and tumor-infiltrating lymphocytes (TILs). ER/PR expression status: Positive expression was defined when nuclear-stained tumor cells accounted for ≥1% of total tumor cells; otherwise, it was negative^1^. HER2 expression status: Positive expression was defined as IHC (3+), or IHC (2+) with FISH (+); IHC (2+) with FISH (-), IHC (1+), and IHC (0) were considered negative for HER2 expression. Molecular subtype: Based on the expression of ER, PR, and HER2, breast cancer was categorized into four molecular subtypes: HR+/HER2-, HR+/HER2+, HR-/HER2+, and HR-/HER2- (TNBC). Ki-67 expression was quantified by the percentage of immunoreactive nuclei in invasive carcinoma cells, with high expression defined as ≥20% and low expression as <20% staining. Stromal TIL density was quantified according to the 2014 recommendations of the International Working Group on TILs in Breast Cancer (9), defined as the percentage of tumor stromal area infiltrated by lymphocytes. TIL levels were stratified as low (<10%) or moderate-to-high (≥10%). pCR was defined as the absence of invasive carcinoma in the primary lesion, with or without DCIS allowed, and negative regional lymph nodes (ypT0/is ypN0).

### MRI acquisition and evaluations

In the pre-neoadjuvant MRI, the assessment included the amount of fibroglandular tissue (FGT; non-dense, dense), the level of background parenchymal enhancement (BPE; minimal or mild, moderate or marked), the morphological features of each lesion, the type of time-signal intensity curve (TIC; increasing/plateau, washout), the signal intensity on T2-weighted imaging (T2WI), associated features, peritumoral edema, subcutaneous edema, and lymph node status. The morphological features included tumor size (maximum tumor diameter), tumor distribution (single, multifocal, or multicentric), lesion type (mass, non-mass enhancement [NME], mass with NME), distribution of NME, internal enhancement of the mass, as well as the shape and margin of the mass. On pre-NAT MRI, the assessment included: radiologic complete remission (rCR), tumor size and type of TIC. Radiologic complete response (rCR) was defined in strict accordance with RECIST 1.1 criteria: (10) the complete absence of both early and late enhancement, and the short-axis diameter of all pathological lymph nodes (whether target or non-target) must be <10 mm. Because the consequences of undertreatment attributable to undetected residual disease outweigh those of overtreatment, we deliberately adopted a more stringent rCR threshold than has been employed in prior reports. This high specificity definition maximizes the avoidance of false-negative classifications but necessarily lowers MRI sensitivity. Consequently, the observed rCR rate in the present study is expected to be lower than that reported in series using conventional criteria. Finally, by comparing the MRI data before and after neoadjuvant therapy, the indexes of variability were derived, including the tumor size change (Δ tumor size) and TIC type change (Δ TIC), where ΔTIC-negative indicates no change and ΔTIC-positive indicates change. Finally, the time intervals between hollow needle biopsy and MRI examination prior to NAT (days to biopsy), as well as between the last MRI examination after NAT and surgery (days to surgery), were retrospectively collected. Breast MRI examinations before and after NAT were independently interpreted by two radiologists with more than five years of experience in breast imaging diagnosis. In addition, both radiologists were blinded to the pathological results. Discordant cases achieved consensus with re-review of the images and discussion.

### Statistical analysis

To evaluate the diagnostic performance of MRI for pCR, rCR was classified as “negative” and non-rCR as “positive.” On this basis, sensitivity, specificity, accuracy, positive predictive value (PPV), and negative predictive value (NPV) were calculated, each with 95% confidence intervals (CIs). The reference definitions were as follows: true negative (TN), MRI indicated rCR and pathology confirmed pCR; true positive (TP), MRI indicated non-rCR and pathology showed non-pCR; false negative (FN), MRI indicated rCR but pathology showed non-pCR; false positive (FP), MRI indicated non-rCR but pathology showed pCR. Patients with FP or FN results were assigned to the imaging–pathology discordant group, whereas those with TP or TN were assigned to the concordant group.

For continuous variables, the Shapiro-Wilk (SW) normality test was first performed. Data meeting the criteria for normal distribution were expressed as mean ± standard deviation (x ± s), and independent samples t-tests or analysis of variance (ANOVA) were used for between-group comparisons; data not following a normal distribution were expressed as the median and interquartile range [M (Q1, Q3)], and the Wilcoxon rank-sum (Mann–Whitney U) tests were used for intergroup comparisons. For categorical data, rates or proportions (%) were used, and Pearson’s chi-square test or Fisher’s exact test was employed for intergroup comparisons. Based on the results of intergroup comparisons, potential influencing factors were preliminarily identified. Further univariate and multivariate logistic regression analyses were conducted to determine independent factors influencing the accuracy of MRI assessment of NAT efficacy. Variables with *p*<0.05 in univariate logistic regression analysis are included in further multivariate analysis.

Patients were randomly allocated to training (70%) and validation (30%) cohorts. Baseline characteristics were compared to ensure cohort balance. Within the training set, multivariable logistic regression identified independent predictors of pCR using surgical pathology as the reference standard. Bootstrap resampling (1000 iterations) was performed to assess the stability of predictors. Variables with confidence intervals excluding zero and sign consistency >90% across bootstrap samples were considered stable. A predictive nomogram was developed and validated for NAT response assessment. The model’s performance was evaluated using the following metrics: discriminative ability by the area under the receiver operating characteristic curve (AUC), calibration by calibration curves, goodness-of-fit by the Brier score, as well as clinical utility by decision curve analysis.

Cohen’s Kappa coefficient was used to assess the agreement of the interpretations of the two radiologists. Interpretation criteria of agreement were as follows: 0.00– 0.20, poor; 0.21–0.40, fair; 0.41–0.60, moderate; 0.61–0.80, substantial; and 0.81–1.00, almost perfect.

All tests with *p*<0.05 were considered statistically significant. Statistical analyses were conducted using SPSS for Windows (version 25.0; SPSS, Inc, Chicago, IL, USA) and R software (version 4.4.3).

## Results

### Clinical and pathological characteristics

A total of 554 patients were enrolled in this study, and the median age at diagnosis was 48 years (interquartile range: 40–57 years). The initial clinical stage was mainly stage I/II (n=354, 63.9%). The molecular subtype distribution: HR+/HER2- 44.9% (n=249), HR-/HER2 + 19.9% (n=110), HR+/HER2 + 19.1% (n=106), and TNBC 16.1% (n=89). All patients received 4–8 cycles NAT. The median interval between the last MRI examination and surgery was 9 days (IQR 3–13 days). Regarding the choice of breast surgery, 86.8% (n=481) underwent mastectomy and 13.2% (n=73) underwent breast-conserving surgery.

### Diagnostic accuracy assessment

Pathological results of post-NAT surgical specimens showed that 136 patients (24.5%) achieved pCR; among 418 (75.5%) non-pCR patients, the median size of paraffin pathological residual tumors was 1.6 cm (interquartile spacing: 0.0-2.5 cm), and 15 breasts had no invasive carcinoma residue but axillary lymph node metastases were present. Post-NAT MRI evaluation showed that only 15 cases (2.7%) achieved rCR; among the 539 non-rCR patients, 301 (55.8%) had mass enhancement, 146 (27.1%) had NME, and 92 (17.1%) had the coexistence of mass lesions and NME. The overall accuracy of breast MRI in assessing the efficacy of NAT was 77.44%. Of the 429 (77.4%) pathologies assessed accurately, pCR and rCR accounted for 3.0% (13 cases) and non-pCR and non-rCR accounted for 97.0% (416 cases); and of the 125 (22.6%) cases assessed inaccurately, non-pCR but rCR accounted for 1.6% (2 cases) and pCR but non-rCR accounted for 98.4% (123 cases), as detailed in **Table 1**.The performance of MRI in diagnosing pCR is as follows: sensitivity 99.5% (95% CI 98.2–99.9%, Wilson method), specificity 9.6% (95% CI 5.6–15.6%, Clopper-Pearson method), PPV 77.2% (95% CI 73.5–80.5%, Wilson method), NPV 86.7% (95% CI 61.1–96.0%, Wilson method).

**Table 1:** Analysis of agreement between MRI and pathological assessment.

		Pathological assessment		Total
		non-pCR	pCR	
MRI assessment	non-rCR	416	123	539
	rCR	2	13	15
Total		418	136	554

### Analysis of influencing factors of MRI in evaluating the accuracy of NAT efficacy

Clinicopathological characteristic analysis showed significant differences between the imaging-pathology consistent group and discordance group in neoadjuvant regimen, neoadjuvant treatment cycles, histological grade, expressions of ER, PR, HER2, Ki-67 (*p* = 0.003), and TILs (*p* = 0.043), as well as presence of DCIS (*p*<0.001, **Table 2**). In the analysis of MRI characteristics, the two groups differed significantly in BPE (*p* = 0.005) and lesion type (*p* = 0.013) (**Table 3**), with no statistical significance found in the remaining indexes (*p >*0.05).

**Table 2:** Comparison of clinicopathological characteristics between concordant and discordant groups.

			Discordant	Concordant	*p* value
			(n=125)	(n=429)	
Clinical characteristics	Age(y)		48[40,57]	48[39,56]	0.774
	Menopausal status	Premenopausal	68(54.4)	260(60.6)	0.214
		Postmenopausal	57(45.6)	169(39.4)	
	Lymph node status	Negative	42(33.6)	111(25.9)	0.089
		Positive	83(66.4)	318(74.1)	
	Clinical stage	I/II	83(66.4)	271(63.2)	0.508
		III	42(33.6)	158(36.8)	
	Neoadjuvant regimens	AT/AC-T	22(17.6)	223(52.0)	<0.001
		HER2-targeted	91(72.8)	112(26.1)	
		Other	12(9.6)	94(21.9)	
	Neoadjuvant treatment cycle		6[6,6]	6[6,6]	<0.001
	Breast surgery type	Mastectomy	110(88.0)	371(86.5)	0.658
		Breast-conserving surgery	15(12.0)	58(13.5)	
Pathologic characteristics	Histologic grade	I/II	63(50.4)	291(67.8)	<0.001
		III	62(49.6)	138(32.2)	
	Histological type	IDC	107(85.6)	363(84.6)	0.787
		Other	18(14.4)	66(15.4)	
	Molecular subtype	HR+/HER2-	8(6.4)	241(56.2)	<0.001
		HR+/HER2+	30(24.0)	76(17.7)	
		HR-/HER2+	64(51.2)	46(10.7)	
		HR-/HER2-	23(18.4)	66(15.4)	
	ER	Negative	87(69.6)	112(26.1)	<0.001
		Positive	38(30.4)	317(73.9)	
	PR	Negative	102(81.6)	168(39.2)	<0.001
		Positive	23(18.4)	261(60.8)	
	HER2	Negative	31(24.8)	307(71.6)	<0.001
		Positive	94(75.2)	122(28.4)	
	Ki-67 Expression	Ki-67<20%	3(2.4)	47(11.0)	0.003
		Ki-67≥20%	122(97.6)	382(89.0)	
	TILs	TILs<10%	101(80.8)	377(87.9)	0.043
		TILs≥10%	24(19.2)	52(12.1)	
	Presence of DCIS	Negative	96(76.8)	253(59.0)	<0.001
		Positive	29(23.2)	176(41.0)	

**Table 3:** Comparison of MRI characteristics between concordant and discordant groups.

			Discordant	Concordant	*p* value
			(n=125)	(n=429)	
	Days to biopsy		2[-1,10]	2[-1,7]	0.500
	Days to surgery		7[3,13]	7[3,14]	0.760
	FGT	Non-dense	109(87.2)	372(86.7)	0.887
		Dense	16(12.8)	57(13.3)	
	BPE	Minimal or Mild	86(68.8)	234(54.5)	0.005
		Moderate or Marked	39(31.2)	195(45.5)	
	Tumor distribution	Single	103(82.4)	368(85.8)	0.351
		Multifocal or multicentric	22(17.6)	61(14.2)	
	Lesion type	Mass	68(54.4)	242(56.4)	0.013
		NME	25(20.0)	122(28.4)	
		Mass and NME	32(25.6)	65(15.2)	
NME	Distribution of NME	Linear or Focal	12(48.0)	63(51.6)	0.952
		Segmental or Regional	10(40.0)	43(35.2)	
		Multiple regional or Diffuse	3(12.0)	16(13.1)	
Mass	Shape of mass	Irregular	68(100.0)	237(97.9)	0.516
		Round or oval	0(0.0)	5(2.1)	
	Margin of mass	Circumscribed	0(0.0)	4(1.7)	0.117
		Not circumscribed	66(97.1)	215(88.8)	
		Spiculated	2(2.9)	23(9.5)	
	Internal enhancement of mass	Heterogenous	66(97.1)	235(97.1)	1.000
		Rim enhancement	2(2.9)	7(2.9)	
	Tumor size(cm)		4.8[3.3,7.0]	4.6[3.2,7.2]	0.753
	T2WI	Low signal	61(48.8)	195(45.5)	0.509
		High signal	64(51.2)	234(54.5)	
	TIC	Increasing/Plateau	10(8.0)	29(6.8)	0.633
		Washout	115(92.0)	400(93.2)	
	Associated features*	Negative	9(7.2)	36(8.4)	0.668
		Positive	116(92.8)	393(91.6)	
	Peritumoral edema	Negative	95(76.0)	346(80.7)	0.256
		Positive	30(24.0)	83(19.3)	

*Associated features include nipple retraction, nipple invasion, skin retraction, skin thickening, skin invasion, axillary adenopathy, pectoralis muscle invasion, chest wall invasion, architectural distortion, and so on.

Univariate logistic regression analysis showed that neoadjuvant regimen, neoadjuvant treatment cycles, histologic grade, expressions of ER, PR, HER2, Ki-67 and TILs, presence of DCIS, BPE, and lesion type were all significantly correlated with the accuracy of the NAT efficacy as assessed by MRI (*p*<0.05). Multivariate logistic regression analysis further revealed that ER-negative (OR = 0.300, 95% CI: 0.152-0.592, *p* = 0.001), absence of DCIS (OR = 0.522, 95% CI: 0.301-0.905, *p* = 0.021), and the coexistence of mass lesions and NME (OR = 0.469, 95% CI: 0.226-0.974, *p* = 0.042) were independent influential factors of inaccurate MRI assessment of NAT efficacy (**Table 4**).

**Table 4:** Univariate and multivariate analysis for factors associated with discordance between MRI and pathological assessment.

		Univariate			Multivariate		
		OR	95%CI	*p* value	OR	95%CI	*p* value
Clinical characteristics	Neoadjuvant regimens			<0.001			0.651
	Other	reference					
	AT/AC-T	0.773	0.367-1.625	0.497	1.999	0.458-8.722	0.357
	HER2-targeted	6.365	3.285-12.332	<0.001	1.055	0.465-2.392	0.898
	treatment cycles	0.644	0.541-0.766	<0.001	0.959	0.778-1.182	0.692
Pathologic characteristics	Histologic grade						
	I/II	reference					
	III	2.075	1.384-3.111	<0.001	0.888	0.530-1.487	0.651
	ER						
	Negative	reference					
	Positive	0.154	0.100-0.239	<0.001	0.3	0.152-0.592	0.001
	PR						
	Negative	reference					
	Positive	0.145	0.089-0.237	<0.001	0.585	0.277-1.235	0.160
	HER2						
	Negative	reference					
	Positive	7.630	4.832-12.050	<0.001	3.11	0.698-13.864	0.137
	Ki-67						
	Ki-67<20%	reference					
	Ki-67≥20%	5.003	1.530-16.362	0.008	1.139	0.297-4.368	0.849
	TILs						
	TILs<10%	reference					
	TILs≥10%	1.723	1.013-2.930	0.045	1.583	0.829-3.020	0.164
	Presence of DCIS						
	Negative	reference					
	Positive	0.434	0.275-0.686	<0.001	0.522	0.301-0.905	0.021
MRI characteristics	BPE						
	Minimal or Mild	reference					
	Moderate or Marked	0.544	0.356-0.831	0.005	0.649	0.391-1.078	0.095
	Lesion type			0.014			0.123
	Mass and NME	reference					
	Mass	0.571	0.346-0.942	0.028	0.645	0.348-1.196	0.164
	NME	0.416	0.228-0.761	0.004	0.469	0.226-0.974	0.042

### Comparison of baseline characteristics between the training cohort and validation cohort

In this study, 388 patients were included in the training cohort and 166 in the validation cohort, and a comparison of the baseline characteristics was shown in **Table 5**. The statistics showed that there were significant differences between the training and validation cohorts in terms of the T2WI signal intensity (*p* = 0.036, φ=0.089), the type of TIC (*p* = 0.003, φ=0.128), and peritumor edema (*p* = 0.005, φ=0.119) on pre-NAT breast MRI, while the rest of the characteristics were not statistically significant (*p*>0.05). After correction by the Bonferroni method (α=0.05/33≈0.0015), these differences were no longer significant. All standardized effect sizes were below 0.3, indicating negligible clinical differences. The baseline characteristics demonstrated satisfactory comparability between cohorts.

**Table 5:** Comparison of baseline characteristics between the training and validation cohorts.

Characteristics				Training cohort	Validation cohort	*P* value
				(n=388)	(n=166)	
Clinical characteristics	Age(y)			48 [39,56]	49 [41,57]	0.166
	Menopausal status		Premenopausal	231(59.5)	97(58.4)	0.809
			Postmenopausal	157(40.5)	69(41.6)	
	Lymph node status		Negative	108(27.8)	45(27.1)	0.861
			Positive	280(72.2)	121(72.9)	
	Clinical stage		I/II	249(64.2)	105(63.3)	0.836
			III	139(35.8)	61(36.7)	
Pathologic characteristics	pCR		Negative	299(77.1)	119(71.7)	0.178
			Positive	89(22.9)	47(28.3)	
	Histologic grade		I/II	250(64.4)	104(62.7)	0.689
			III	138(35.6)	62(37.3)	
	Histological type		IDC	332(85.6)	138(83.1)	0.464
			Other	56(14.4)	28(16.9)	
	Molecular subtype		HR+/HER2-	179(46.1)	70(42.2)	0.287
			HR+/HER2+	66(17.0)	40(24.1)	
			HR-/HER2+	79(20.4)	31(18.7)	
			HR-/HER2-	64(16.5)	25(15.1)	
	ER		Negative	143(36.9)	56(33.7)	0.483
			Positive	245(63.1)	110(66.3)	
	PR		Negative	190(49.0)	80(48.2)	0.867
			Positive	198(51.0)	86(51.8)	
	HER2		Negative	244(62.9)	94(56.6)	0.166
			Positive	144(37.1)	72(43.4)	
	Ki-67 expression		Ki-67<20%	31(8.0)	19(11.4)	0.193
			Ki-67≥20%	357(92.0)	147(88.6)	
	TILs		TILs≤10%	339(87.4)	139(83.7)	0.254
			TILs>10%	49(12.6)	27(16.3)	
	Presence of DCIS		Negative	236(60.8)	113(68.1)	0.106
			Positive	152(39.2)	53(31.9)	
Pre-NAT MRI characteristics	Lymph node status		Negative	77(19.8)	26(15.7)	0.246
			Positive	311(80.2)	140(84.3)	
	FGT		Non-dense	330(85.1)	151(91.0)	0.059
			Dense	58(14.9)	15(9.0)	
	BPE		Minimal or Mild	230(59.3)	90(54.2)	0.269
			Moderate or Marked	158(40.7)	76(45.8)	
	Tumor distribution		Single	334(86.1)	137(82.5)	0.283
			Multifocal or multicentric	54(13.9)	29(17.5)	
	Lesion type		Mass	223(57.5)	87(52.4)	0.538
			NME	100(25.8)	47(28.3)	
			Mass and NME	65(16.8)	32(19.3)	
	NME	Distribution of NME	Linear or Focal	47(47.0)	28(59.6)	0.187
			Segmental or Regional	37(37.0)	16(34.0)	
			Multiple regional or Diffuse	16(16.0)	3(6.4)	
	Mass	Shape of mass	Irregular	220(98.7)	85(97.7)	0.923
			Round or oval	3(1.3)	2(2.3)	
		Margin of mass	Circumscribed	2(0.9)	2(2.3)	0.634
			Not circumscribed	203(91.0)	78(89.7)	
			Spiculated	18(8.1)	7(8.0)	
		Internal enhancement of mass	Heterogenous	217(97.3)	84(96.6)	1.000
			Rim enhancement	6(2.7)	3(3.4)	
	Tumor size			4.5[3.1,7.2]	4.9[3.5,7.0]	0.340
	T2WI		Low signal	168(43.3)	88(53.0)	0.036
			High signal	220(56.7)	78(47.0)	
	TIC		Increasing/ Plateau	19(4.9)	20(12.0)	0.003
			Washout	369(95.1)	146(88.0)	
	Peritumoral edema		Negative	321(82.7)	120(72.3)	0.005
			Positive	67(17.3)	46(27.7)	
	subcutaneous edema		Negative	326(84.0)	142(85.5)	0.651
			Positive	62(16.0)	24(14.5)	
Post-NAT MRI characteristics	rCR		Negative	379(97.7)	160(96.4)	0.566
			Positive	9(2.3)	6(3.6)	
	Tumor size			3.3[2.2,4.8]	3.3[2.3,5.0]	0.520
	TIC		Increasing/ Plateau	282(72.7)	128(77.1)	0.276
			Washout	106(27.3)	38(22.9)	
	ΔTIC		Negative	108(27.8)	36(21.7)	0.131
			Positive	280(72.2)	130(78.3)	
	ΔTumor size			1.1[0.5,1.8]	1.1[0.5,2.2]	0.492

### Analysis of influencing factors of the efficacy of neoadjuvant therapy

Univariable regression analysis (**Table 6**) identified several factors associated with pCR (*p* < 0.05), including lymph node status, histologic grade, expression of ER, PR, HER2, Ki-67, and TILs, presence of DCIS, BPE, lesion type, and peritumoral edema on pre-NAT breast MRI, rCR, tumor size, TIC on post-NAT breast MRI, as well as ΔTumor size and ΔTIC.

**Table 6:** Univariate and multivariate analyses of factors associated with pCR.

Characteristics		Univariate			Multivariate		
		OR	95%CI	P value	OR	95%CI	P value
Clinical characteristics	Lymph node status						
	Negative	reference					
	Positive	0.643	0.424-0.976	0.038	0.715	0.404-1.266	0.249
	Histologic grade						
	I/II	reference					
	III	2.256	1.521-3.348	<0.001	1.144	0.652-2.008	0.640
	ER						
	Negative	reference					
	Positive	0.157	0.103-0.240	<0.001	0.249	0.119-0.520	<0.001
	PR						
	Negative	reference					
	Positive	0.138	0.086-0.223	<0.001	0.574	0.261-1.262	0.167
	HER2						
	Negative	reference					
	Positive	8.425	5.385-13.180	<0.001	6.041	3.388-10.771	<0.001
	Ki-67						
	Ki-67<20%	reference					
	Ki-67≥20%	8.692	2.084-36.256	0.003	2.807	0.515-15.287	0.233
	TILs						
	TILs≤10%	reference					
	TILs>10%	1.998	1.196-3.338	0.008	1.471	0.732-2.955	0.279
	Presence of DCIS						
	Negative	reference					
	Positive	0.373	0.237-0.587	< 0.001	0.495	0.273-0.899	0.021
Pre-NAT MRI characteristics	BPE						
	Minimal or Mild	reference					
	Moderate or Marked	0.599	0.399-0.899	0.013	0.585	0.335-1.022	0.060
	Lesion type			0.005			0.058
	Mass and NME	reference					
	Mass	0.565	0.347-0.922	0.022	0.45	0.207-0.981	0.045
	NME	0.381	0.210-0.688	0.001	0.415	0.187-0.923	0.031
	Peritumoral edema						
	Negative	reference					
	Positive	1.594	1.012-2.511	0.044	0.795	0.417-1.514	0.485
post-NAT MRI characteristics	rCR						
	Negative	reference					
	Positive	21.984	4.894-98.744	< 0.001	19.888	2.985-132.498	0.002
	Tumor size	0.888	0.806-0.980	0.018	0.78	0.662-0.920	0.003
	TIC						
	Increasing/Plateau	reference					
	Washout	0.077	0.031-0.191	< 0.001	0.033	0.005-0.240	0.001
	ΔTumor size	1.260	1.100-1.442	0.001	1	0.821-1.217	0.996
	ΔTIC						
	Negative	reference					
	Positive	7.716	3.670-16.222	< 0.001	0.363	0.062-2.131	0.262

Multivariable logistic regression analysis revealed that ER-negative status (OR, 0.249 [95% CI: 0.119–0.520]; *p*<0.001), HER2-positive status (OR, 6.041 [95% CI: 3.388–10.771]; *p*<0.001), absence of DCIS (OR, 0.495 [95% CI: 0.273–0.899]; *p* = 0.021), rCR (OR, 19.888 [95% CI: 2.985–132.498]; *p* = 0.002), smaller tumor size (OR, 0.780 [95% CI: 0.662–0.920]; *p* = 0.003), and increasing/plateau TIC (OR, 0.033 [95% CI: 0.005–0.240]; *p* = 0.001) were associated with a higher pCR rate. On pre-NAT MRI, mass lesions (OR = 0.45) and NME (OR = 0.415) were associated with a lower pCR rate compared to Mass and NME mixed lesions.

Meanwhile, we performed an additional 1,000 bootstrap resampling iterations in the training set to evaluate variable stability. As a result, the 95% confidence interval for the coefficient of the lesion type variable included zero, and this variable exhibited low sign consistency (Mass type: 50.2%; NME type: 89.6%; **Supplementary Table 7**). Consequently, the lesion type variable was excluded from the final model. The final multivariable model incorporated six robust predictor variables (**Supplementary Table 8**). Subsequent internal validation via bootstrap resampling confirmed excellent stability for all included variables, with 95% confidence intervals excluding zero and sign consistency exceeding 99% for every predictor.

### Development and validation of the nomogram

**Figure 2:**
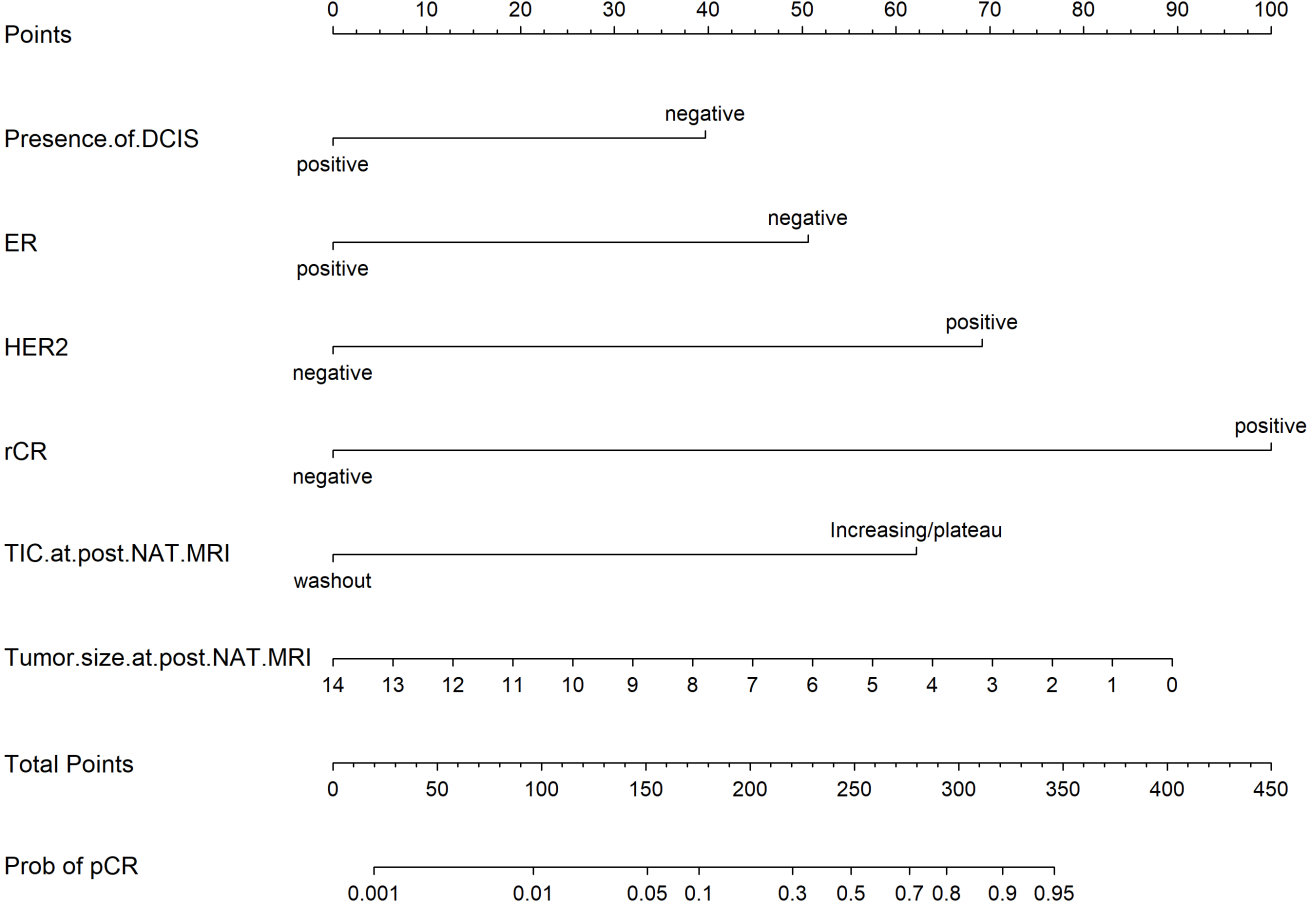
Predictive nomogram for pCR probability.

**Figure 3:**
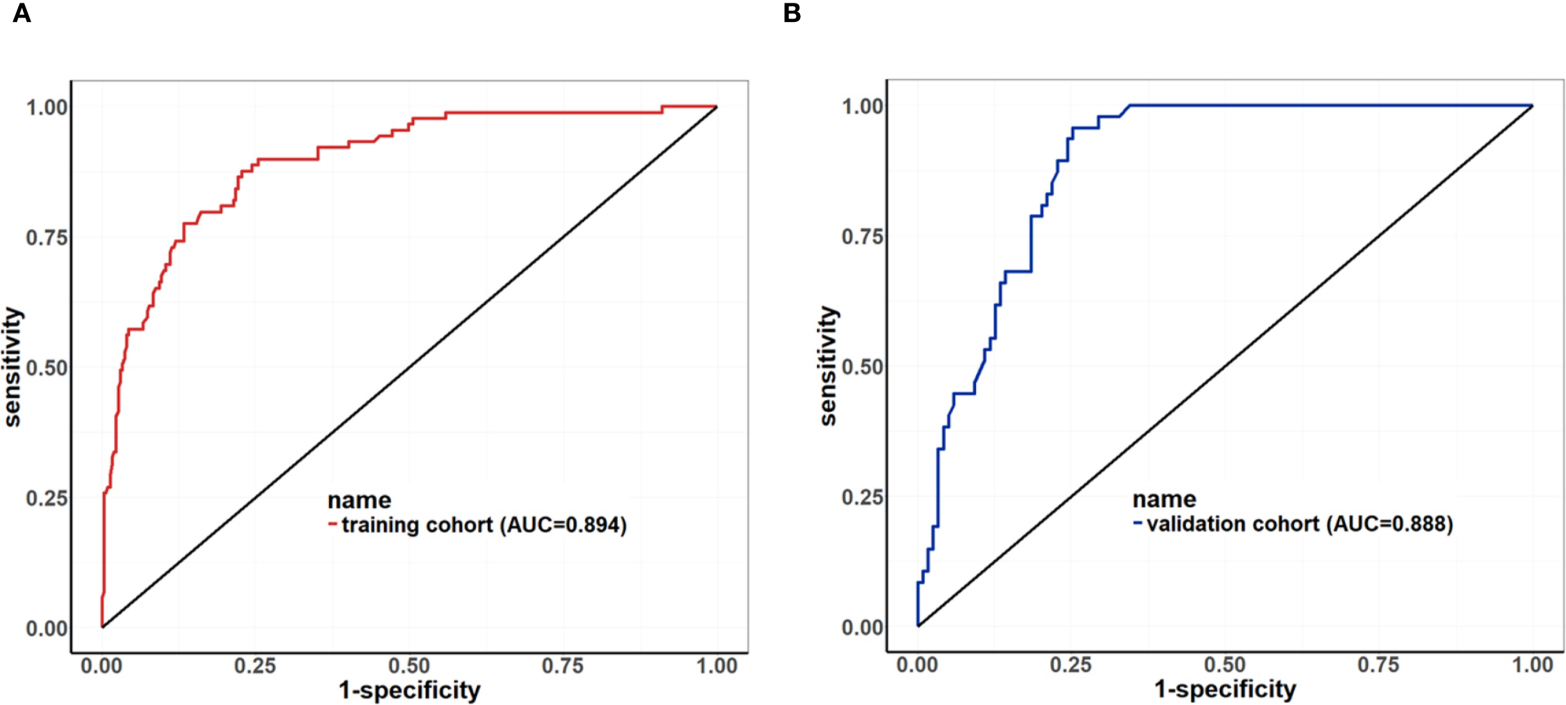
ROC Curve of the nomogram for predicting pCR. **(A)** ROC of the training cohort (AUC = 0.894, 95% CI: 0.857–0.932); **(B)** ROC of the validation cohort (AUC = 0.888, 95% CI: 0.841–0.935).

**Figure 4:**
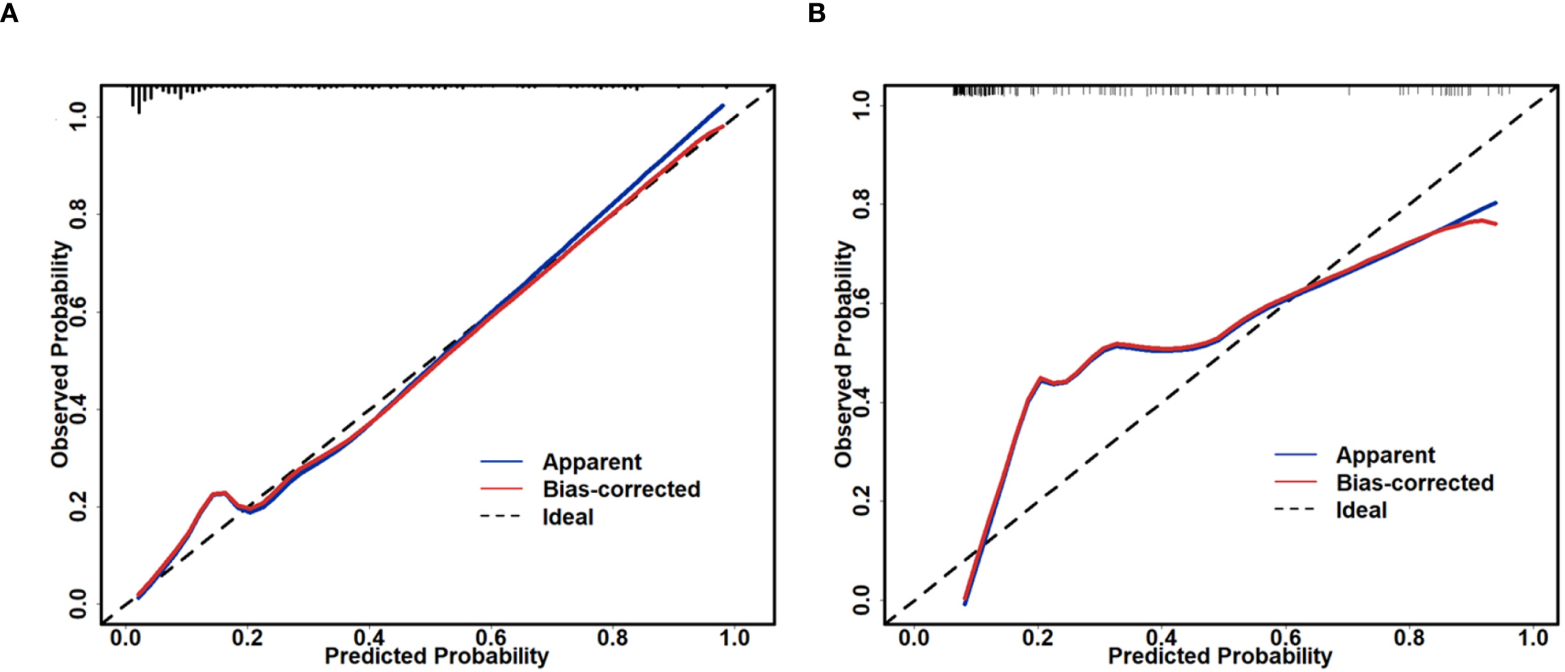
Calibration plot of the nomogram for predicting pCR. **(A)** calibration plot of the training cohort; **(B)** calibration plot of the validation cohort.

**Figure 5:**
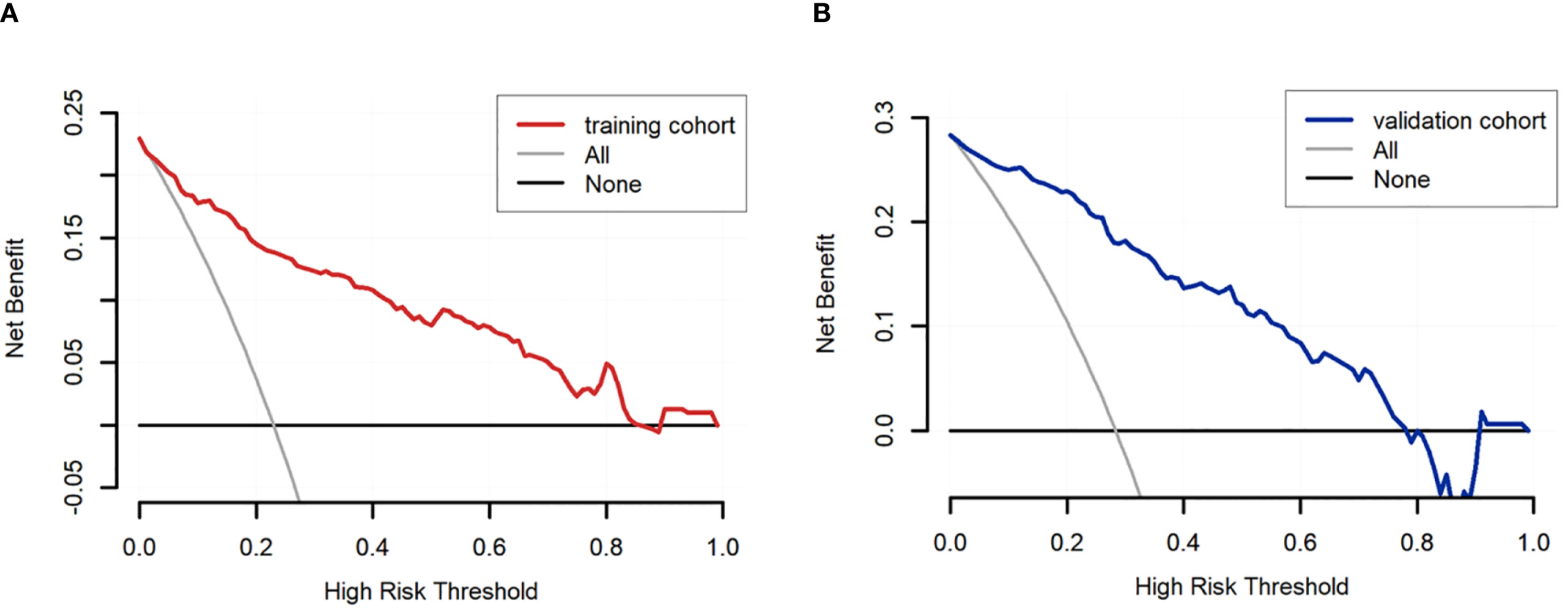
DCA of the nomogram for predicting pCR. **(A)** DCA curve of the training cohort; **(B)** DCA curve of the validation cohort.

Based on the univariate and multivariate Logistic regression analyses of the development cohort, a nomogram model for predicting pCR was further constructed (**Figure 2**). The nomogram demonstrated good discrimination ability in both the training cohort (AUC = 0.894, 95% CI: 0.857–0.932) (**Figure 3A**) and the validation cohort (AUC = 0.888, 95% CI: 0.841–0.935) (**Figure 3B**). Model calibration was deemed acceptable. In the training cohort, the calibration curve fitted well with the ideal calibration line (**Figure 4A**), and in the validation cohort, the calibration curve still generally clustered around the ideal calibration line (**Figure 4B**). The Brier scores for the training and validation cohorts were 0.102 and 0.131, respectively. Finally, the clinical decision curve showed (**Figures 5A, B**) that the net benefit of the model was better than that of the all-pCR/all-non-pCR diagnostic strategy at most thresholds, and it has clinical application value.

## Discussion

Data from our study indicated an overall accuracy of 77.44% for breast MRI in assessing the response to NAT, consistent with previous research (11). Investigating the factors contributing to radiologic-pathologic discordance will facilitate optimized clinical interpretation of MRI findings in patients with specific clinicopathological or imaging characteristics, thereby providing a basis for enhancing MRI performance.

Current studies on the impact of concomitant DCIS on MRI accuracy predominantly rely on postoperative paraffin-embedded pathology, suggesting reduced MRI assessment accuracy in patients with DCIS—a finding influenced by pCR definitions (12, 13). In contrast, this study focuses on early assessment of NAT response, addressing the inherent time lag of conventional pathological evaluation. Notably, we observed higher MRI inaccuracy in patients without DCIS on core needle biopsy, a finding discordant with conventional understanding. We postulate this discrepancy may stem from sampling limitations of core needle biopsy, wherein multifocal or microscopic DCIS foci could remain undetected (14). In cases diagnosed via core needle biopsy as “without concomitant DCIS,” there may exist diffusely distributed DCIS components that were not sampled during the biopsy procedure. Such occult DCIS tends to be less responsive to NAT and is more likely to persist post-treatment. Its persistent enhancement on MRI may be misinterpreted as residual invasive carcinoma, thereby reducing the specificity and overall accuracy of MRI assessment in this subgroup. In contrast, the classification of cases with biopsy-confirmed “concomitant DCIS” is more reliable. Therefore, in clinical practice, caution should be exercised regarding the risk of false-positive MRI findings due to residual DCIS after NAT in patients initially diagnosed as DCIS-negative on biopsy. These conclusions still require further validation through large-scale prospective studies. Consequently, these results warrant cautious interpretation and further validation. The observation presents dual challenges for breast imaging and pathology. Future research may explore quantitative analysis of multimodal MRI and artificial intelligence imaging recognition techniques, in combination with more precise biopsy strategies (e.g., stereotactic localization, multi-target sampling), to further enhance the accuracy of MRI assessment.

In this study, it was found that the diagnostic efficacy of breast MRI varies among different molecular subtypes of breast cancer. The accuracy rates in each subtype are as follows: HR+/HER2- type (96.79%), HR-/HER2- type (74.16%), HR+/HER2+ type (67.92%), and HR-/HER2+ type (41.82%). These findings are generally consistent with the trends reported in previous literature, which are 94.5% for Luminal A type, 74.4% for Luminal B type, 88.9% for TNBC, and 58.2% for HER2 type (7). The relatively high accuracy of MRI assessment in TNBC may be directly related to its unique cellular and vascular characteristics. TNBC typically exhibits a higher histological grade, accompanied by increased cellular proliferation, higher cellular density, greater structural complexity, and significantly enhanced metabolic activity. Furthermore, it demonstrates increased neovascularization, leading to aberrant tumor vascular structure and function, elevated arteriovenous shunting, and dysregulated blood flow. These features collectively promote greater uptake of contrast agent and enhanced leakage into the extracellular space, ultimately manifesting as more pronounced enhancement on MRI. This likely underlies the higher diagnostic accuracy of MRI for TNBC (15, 16). Conversely, the lower MRI diagnostic accuracy for HER2-positive tumors may be mechanistically explained by several factors. Firstly, HER2 gene amplification is closely linked to neovascularization. Targeted therapies inhibit HER2 signaling transduction, disrupting the balance between pro-angiogenic and anti-angiogenic factors. This suppresses angiogenesis and promotes the normalization of abnormal vasculature, consequently altering MRI imaging characteristics (17). Secondly, the residual effects of angiogenesis may play a role. HER2-positive breast cancers are inherently highly proliferative and often present with a high level of angiogenesis at diagnosis. Even after achieving a pCR following NAT, residual neovasculature may still manifest as a non-rCR on MRI, contributing to reduced diagnostic accuracy (18).

Regarding baseline MRI features, multiple studies have demonstrated their association with MRI-pathology discordance following NAT. Research by Hu et al. indicated that multifocal/multicentric lesions, segmental or regional NME distribution, and enhancing mass margins increase the risk of discordance (7). Negrão et al. reported that NME was the sole significant factor associated with MRI-pathology discordance (19). The reduced accuracy of MRI assessment for NME lesions stems from two primary factors. Firstly, unlike well-defined, homogeneous mass lesions, NME exhibits diffuse, heterogeneous growth patterns and often regresses irregularly after NAT. In contrast, masses typically demonstrate concentric shrinkage. This difference renders MRI size estimation more challenging for NME (20–22). Secondly, focal, regional, or asymmetric background parenchymal enhancement (BPE) can be readily misinterpreted as NME. For instance, Chikarmane et al. found that 20% (77 cases) of lesions previously classified as NME were actually BPE (23). Furthermore, this study revealed that the coexistence of mass and NME components significantly amplifies MRI assessment inaccuracy. This arises from the complex interplay of heterogeneous enhancement patterns, conflicting imaging characteristics, divergent underlying pathology, and variable treatment responses.

The association between the presence of DCIS on core needle biopsy and pCR to NAT remains inconsistent across existing studies. Labrosse et al. found no significant association between concomitant DCIS on biopsy and pCR (14). Conversely, von Minckwitz et al. reported that the absence of DCIS was an independent predictor of pCR in HER2-positive breast cancer (24). Similarly, Helal et al. demonstrated a significant association between the absence of DCIS on biopsy and pCR in TNBC (25). In the present study, we observed comparable results: the absence of DCIS on pre-treatment core biopsy was associated with a higher likelihood of achieving pCR. This observation may be explained by the fact that while DCIS can exhibit some response to NAT and may be completely eradicated in some cases, DCIS cells generally possess lower proliferative and invasive potential compared to invasive carcinoma. Consequently, breast cancers associated with DCIS typically exhibit lower overall response rates to NAT than pure invasive carcinomas, resulting in reduced responsiveness to neoadjuvant treatment (26, 27).

Furthermore, we found that MRI-assessed rCR was significantly correlated with pCR. This aligns with Santamaría et al., who demonstrated that the absence of late-phase enhancement on post-NAT MRI significantly correlated with pCR (28). Similarly, Kim et al. showed that the absence of both early and late enhancement within the tumor bed on post-NAT MRI was independently associated with pCR (29). In our study, rCR was defined as the absence of both early and late enhancement at the primary site on post-treatment MRI, combined with a short-axis diameter of <10 mm for all pathological lymph nodes (targeted or non-targeted). Multivariate regression analysis confirmed rCR as a strong predictor of NAT response. However, due to the limited sample size of rCR cases in this cohort (n=15, 2.7%), future studies with larger cohorts are warranted to validate this finding. Additionally, the post-NAT TIC type was significantly associated with pCR, consistent with previous reports (30). TIC reflects lesion hemodynamics, specifically related to tissue blood flow perfusion and microvascular permeability (31). Patients achieving pCR often exhibit TIC curves characterized as persistent or plateau types. This pattern likely results from tumor vascular remodeling or obliteration, reduced angiogenesis coupled with increased destruction, and decreased vascular wall permeability. Although some literature suggests that changes in TIC pattern between pre- and post-NAT MRI correlate with pCR (32), our multivariate analysis did not reveal a statistically significant association between TIC pattern change and pCR.

Numerous studies have developed predictive models for NAT response based on MRI features (33, 34). Compared to these previous models, the model constructed in this study demonstrated superior performance in terms of discrimination, calibration, and clinical decision applicability. Our present model incorporates only baseline characteristics and MRI parameters on post-NAT breast MRI. Consequently, its primary utility lies in pre-operative risk stratification to inform surgical decision-making. To this end, we systematically evaluated a range of probability thresholds and report the corresponding sensitivities and specificities (**Supplementary Table 9**). To minimize the risk of long-term survival detriment attributable to under-treatment while maintaining adequate sensitivity, we adopted a probability threshold of 0.70. At this threshold, the model achieves a specificity of 97.7% and a sensitivity of 40.4%, thereby correctly identifying the vast majority of patients with pathologically confirmed pCR. Based on this threshold, when the predicted probability is ≥0.70, breast-conserving surgery (BCS) combined with sentinel lymph node biopsy (SLNB) may be discussed with the patient (One representative application case is depicted in the **Supplementary Figure 1**), and the feasibility of omitting surgery could be evaluated in future prospective trials. Conversely, if the predicted probability is <0.70, standard modified radical mastectomy or conventional BCS is recommended.

However, this study has several limitations. First, it was a single-center, retrospective analysis. Second, due to the limited sample size, subgroup analyses based on molecular subtypes were not performed. Additionally, the small number of cases achieving MRI-assessed rCR (n=15, 2.7%) compromised the evaluation of diagnostic performance for this outcome. Finally, the training and validation cohorts exhibited minor discrepancies in a few baseline MRI features. Although statistical analyses and model performance metrics suggest these differences had limited impact, they nevertheless represent a limitation. Future work will undertake external validation in a more independent and balanced cohort to further confirm the model’s generalizability.

## Conclusions

In summary, our research indicates that breast MRI demonstrates good accuracy in predicting NAT response. However, its accuracy in post-NAT efficacy assessment decreases in tumors that are ER-negative, lack concomitant DCIS, and exhibit both mass and non-mass enhancement lesions. Consequently, when utilizing MRI to evaluate NAT response, a comprehensive analysis integrating baseline clinicopathological characteristics and MRI findings is essential. The MRI feature-based predictive model developed here shows promise in efficacy prediction and may serve as a valuable supplementary tool for clinical decision-making.

## Data Availability

The original contributions presented in the study are included in the article/**Supplementary Material**. Further inquiries can be directed to the corresponding authors.

## Supplementary material

Supplementary Figure 1 A representative case from our retrospective cohort notably demonstrates the potential clinical utility of this strategy. A 65-year-old female patient was diagnosed via core needle biopsy with HR-negative/HER2-positive invasive ductal carcinoma (histologic grade 2). Initial staging indicated a tumor measuring 4.5 cm in maximum diameter with lymph node involvement (cT2N1, stage IIb). Key variables incorporated into the model included: DCIS component, ER negativity, HER2 positivity, post-NAT MRI indicating rCR, TIC showing wash-out pattern, and residual tumor size of 0 cm. The nomogram total score was approximately 310, corresponding to a predicted pCR probability exceeding 0.80. Based on the predefined decision threshold of 0.70, the patient was recommended for and successfully underwent breast-conserving surgery combined with sentinel lymph node biopsy. Final pathological evaluation confirmed pCR. This case supports the feasibility of safely omitting further surgery in patients with a nomogram-predicted pCR probability ≥ 0.70 in future prospective trials.
