# Supplementary figures and images for: Personalized prediction of pathological complete response in breast cancer neoadjuvant therapy: a nomogram combining quantitative MRI biomarkers and molecular subtypes
Source: Front Oncol. 2025 Sep 25;15:1669700. doi: 10.3389/fonc.2025.1669700 (PMC12507605; doi:10.3389/fonc.2025.1669700)

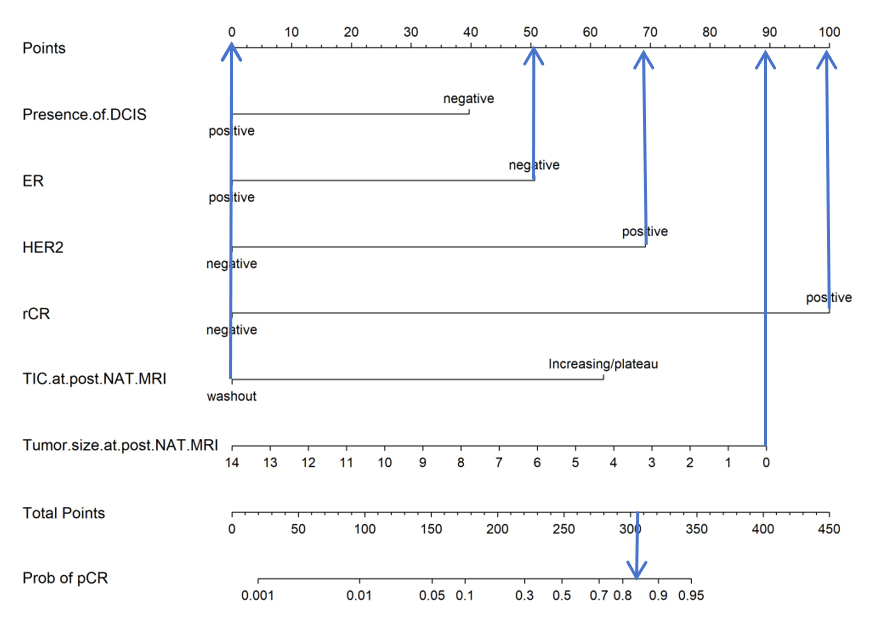

Supplement: Supplementary Figure 1 — A representative case from our retrospective cohort notably demonstrates the potential clinical utility of this strategy. A 65-year-old female patient was diagnosed via core needle biopsy with HR-negative/HER2-positive invasive ductal carcinoma (histologic grade 2). Initial staging indicated a tumor measuring 4.5 cm in maximum diameter with lymph node involvement (cT2N1, stage IIb). Key variables incorporated into the model included: DCIS component, ER negativity, HER2 positivity, post-NAT MRI indicating rCR, TIC showing wash-out pattern, and residual tumor size of 0 cm. The nomogram total score was approximately 310, corresponding to a predicted pCR probability exceeding 0.80. Based on the predefined decision threshold of 0.70, the patient was recommended for and successfully underwent breast-conserving surgery combined with sentinel lymph node biopsy. Final pathological evaluation confirmed pCR. This case supports the feasibility of safely omitting further surgery in patients with a nomogram-predicted pCR probability ≥ 0.70 in future prospective trials. [file Image1.jpeg]
