# Supplementary material for: Personalized prediction of pathological complete response in breast cancer neoadjuvant therapy: a nomogram combining quantitative MRI biomarkers and molecular subtypes
Source: Front Oncol. 2025 Sep 25;15:1669700. doi: 10.3389/fonc.2025.1669700 (PMC12507605; doi:10.3389/fonc.2025.1669700)
Supplement: Supplementary file 2 [file Table1.docx]

**Supplementary Table 7.** Bootstrap analysis of predictor stability in the training cohort (n=1,000 iterations)

| **Predictor** | **Median Coefficient Estimate** | **Median OR** | **95% Confidence Interval** | **Sign Consistency (%)** |
| --- | --- | --- | --- | --- |
| **Presence of DCIS** | -1.192 | 0.30 | (-2.17, -0.38) | 99.8 |
| **ER** | -1.621 | 0.20 | (-2.34, -0.94) | 100.0 |
| **HER2** | 2.113 | 8.27 | (1.37, 2.97) | 100.0 |
| **Lesion Type (Mass)** | -0.002 | 1.00 | (-0.96, 1.02) | 50.2 |
| **Lesion Type (NME)** | 0.729 | 2.07 | (-0.39, 1.81) | 89.6 |
| **rCR** | 2.981 | 19.71 | (0.79, 19.00) | 99.6 |
| **TIC at Post-NAT MRI** | -1.997 | 0.14 | (-3.79, -0.94) | 100.0 |
| **Tumor Size at Post-NAT MRI** | -0.240 | 0.79 | (-0.47, -0.03) | 98.6 |
