# Supplementary material for: Personalized prediction of pathological complete response in breast cancer neoadjuvant therapy: a nomogram combining quantitative MRI biomarkers and molecular subtypes
Source: Front Oncol. 2025 Sep 25;15:1669700. doi: 10.3389/fonc.2025.1669700 (PMC12507605; doi:10.3389/fonc.2025.1669700)
Supplement: Supplementary file 3 [file Table2.docx]

**Supplementary Table 8.** Bootstrap stability validation of predictors in the final multivariable model (n=1,000 iterations).

| **Predictor** | **Median Coefficient Estimate** | **Median OR** | **95% Confidence Interval** | **Sign Consistency (%)** |
| --- | --- | --- | --- | --- |
| **Presence of DCIS** | **-1.217** | **0.30** | **(-2.11, -0.44)** | **99.8** |
| **ER** | **-1.625** | **0.20** | **(-2.32, -0.94)** | **100** |
| **HER2** | **2.156** | **8.64** | **(1.42, 2.96)** | **100** |
| **rCR** | **3.178** | **23.99** | **(0.90, 19.07)** | **99.6** |
| **TIC at Post-NAT MRI** | **-1.946** | **0.14** | **(-3.62, -0.93)** | **100** |
| **Tumor Size at Post-NAT MRI** | **-0.200** | **0.82** | **(-0.37, -0.04)** | **99.3** |
