# Supplementary material for: Personalized prediction of pathological complete response in breast cancer neoadjuvant therapy: a nomogram combining quantitative MRI biomarkers and molecular subtypes
Source: Front Oncol. 2025 Sep 25;15:1669700. doi: 10.3389/fonc.2025.1669700 (PMC12507605; doi:10.3389/fonc.2025.1669700)
Supplement: Supplementary file 4 [file Table3.docx]

**Supplementary Table 9**. Diagnostic performance of different probability thresholds for the nomogram in predicting pCR

| Threshold | Sensitivity | Specificity |
| --- | --- | --- |
| 0.4 | 0.719 | 0.890 |
| 0.5 | 0.573 | 0.933 |
| 0.6 | 0.494 | 0.970 |
| 0.7 | 0.404 | 0.977 |
| 0.8 | 0.258 | 0.997 |
| 0.9 | 0.056 | 1.000 |
