# Supplementary material for: Personalized prediction of pathological complete response in breast cancer neoadjuvant therapy: a nomogram combining quantitative MRI biomarkers and molecular subtypes
Source: Front Oncol. 2025 Sep 25;15:1669700. doi: 10.3389/fonc.2025.1669700 (PMC12507605; doi:10.3389/fonc.2025.1669700)
Supplement: Supplementary file 5 [file Table4.docx]

**Supplementary Table 10.** Comparison of standard logistic regression and Firth's penalized likelihood regression coefficients (sensitivity analysis)

| Characteristics | | | Standard logistic regression | | | |  | Firth’s penalised likelihood logistic regression | | |
| --- | --- | --- | --- | --- | --- | --- | --- | --- | --- | --- |
|  |  |  | OR | 95%CI | p value | |  | OR | 95%CI | p value |
| Clinical characteristics |  | |  |  |  | |  |  |  |  |
|  | ER | |  |  |  | |  |  |  |  |
|  | Negative | | reference |  |  | |  |  |  |  |
|  | Positive | | 0.217 | 0.115-0.407 | ＜0.001 | |  | 0.227 | 0.120-0.417 | ＜0.001 |
|  | HER2 | |  |  |  | |  |  |  |  |
|  | Negative | | reference |  |  | |  |  |  |  |
|  | Positive | | 8.080 | 4.105-15.903 | ＜0.001 | |  | 7.618 | 3.991-15.153 | ＜0.001 |
|  | Presence of DCIS | |  |  | |  |  |  |  |  |
|  | Negative | | reference |  | |  |  |  |  |  |
|  | Positive | | 0.302 | 0.146-0.623 | | 0.001 |  | 0.315 | 0.151-0.629 | ＜0.001 |
| post-NAT MRI characteristics | | rCR |  |  | |  |  |  |  |  |
|  |  | Negative | reference |  | |  |  |  |  |  |
|  |  | Positive | 20.479 | 1.986-211.164 | | 0.011 |  | 14.560 | 2.272-164.880 | 0.004 |
|  |  | Tumor size | 0.825 | 0.703-0.967 | | 0.018 |  | 0.831 | 0.707-0.968 | 0.017 |
|  |  | TIC |  |  | |  |  |  |  |  |
|  |  | Increasing/Plateau | reference |  | |  |  |  |  |  |
|  |  | Washout | 0.153 | 0.054-0.437 | | ＜0.001 |  | 0.170 | 0.056-0.432 | ＜0.001 |
